# Mammilliothalamic Tract Lesions Disrupt Tests of Visuo-Spatial Memory

**DOI:** 10.1037/bne0000001

**Published:** 2014-06-23

**Authors:** Andrew J. D. Nelson, Seralynne D. Vann

**Affiliations:** 1 School of Psychology, Cardiff University

**Keywords:** mammilliothalamic tract, mammillary bodies, anterior thalamic nucleus, spatial memory, recognition memory

## Abstract

The mammillary bodies and their projections via the mammilliothalamic tract to the anterior thalamic nuclei are known to be important for spatial memory in rodents, but their precise role remains unclear. To determine whether transection of the mammilliothalamic tract can produce deficits on tests of spatial memory even when the navigational demands placed on the animal are limited, rats with discrete mammilliothalamic tract lesions were tested on the ability to use distal visual cues to discriminate between 2 locations within a room, irrespective of the direction traveled (Experiment 1). Animals with mammilliothalamic tract lesions acquired this task more slowly and less accurately than control animals. Consistent with this impairment in discriminating different spatial locations, the same lesions also severely disrupted object-in-place memory but spared performance on standard tests of object recognition memory (Experiment 2). Finally, to compare performance on a task that is known to be sensitive to mammilliothalamic transection and requires animals to actively navigate within their environment, the effect of the lesions on spatial working memory in the radial-arm maze was examined. Taken together, the results suggest that even when there are little or no navigational demands, mammilliothalamic tract damage still results in impoverished encoding of spatial location.

The Delay and Brion circuit links an array of interconnected temporal lobe and diencephalic regions, including the hippocampus, fornix, mammillary bodies, anterior thalamus and retrosplenial cortex, that are all heavily implicated in memory (Aggleton & Brown, 1999; Delay & Brion, 1969). The mammilliothalmic tract (MTT), the major efferent of the mammillary bodies to the anterior thalamic nuclei, has a privileged position within this network because it is the only anatomical structure whose connections are restricted to this circuit (Cruce, 1975; Seki & Zyo, 1984). As such, transecting this tract may prove particularly informative for our understanding of the functional significance of the Delay and Brion circuit and, more specifically, interactions between the mammillary bodies and the anterior thalamic nuclei. Its significance is further highlighted by recent evidence demonstrating that disconnection of the hippocampal inputs to the mammillary bodies only results in mild deficits on tests of spatial memory (Vann, Erichsen, O’Mara, & Aggleton, 2011; Vann, 2013). These findings suggest that the mammillary bodies, and their main projections via the MTT, play a crucial role in memory that is independent of its hippocampal inputs (e.g., Vann, 2010; Vann, 2013; Vann & Albasser, 2009; Vann et al., 2011).

Previous investigations into the functional significance of the MTT have shown that lesions to this tract in rats result in a pattern of findings consistent with an impairment in the encoding of spatial information and, in particular, the effective use of extramaze spatial cues (Field, Rosenstock, King, & Greene, 1978; Thomas & Gash, 1985; Vann, 2013; Vann & Aggleton, 2003). However, in these maze-based tasks it is not possible to dissociate navigational impairments from deficits in the effective use of allocentric spatial information. Indeed, it has also been shown that MTT lesions disrupt dead reckoning, which depends on the ability to use self-movement cue processing to estimate the direction and distance to the point where movement originated (Winter, Wagner, McMillin, & Wallace, 2011). One obvious interpretation, which could unite these different findings, is that MTT lesion-induced deficits on spatial tasks reflect a navigational impairment. The aim of the current set of experiments was to characterize further the nature of the spatial memory deficit associated with MTT transection and, more specifically, to determine whether “spatial” deficits could be observed when the navigational demands were limited. To this end, we tested rats with MTT lesions on a series of tasks that required the animals to use spatial information to guide behavior without the need to navigate effectively or monitor where it had traveled within any given trial. The first task taxed the ability to discriminate between two locations within the same room, irrespective of the direction faced (“Location” task, Experiment 1). Rats were trained on a go/no-go discrimination in which they were only reinforced for digging when in the correct location. As direction of travel was irrelevant to solving this discrimination (see Figure 1A), the task assessed the use of distal visual cues in the absence of any obvious navigational component. If the MTT lesions selectively disrupt the processing of self-movement cues, then the MTT leisoned animals should acquire this discrimination at an equivalent rate to control animals. Conversely, if the MTT has a broader role in spatial cognition that includes the encoding of environmental cues, then MTT transection would be expected to impair acquisition of this location discrimination. In order to test whether go/no-go procedures are appropriate for rats with MTT lesions, the rats were initially trained on a nonspatial go/no-go task involving the discrimination of distinct digging media, where only one media consistently contained food. Subsequently, the effect of MTT lesions on object-in-place memory was assessed (Experiment 2). Tests of object-in-place memory not only examine rats’ ability to maintain the features of an object but also the spatial location in which the object was first encountered (Dix & Aggleton, 1999). Performance on this task was contrasted with standard tests of object recognition in which there is no spatial element. If the MTT has a general role in the encoding of spatial location, then the MTT lesion animals should selectively be impaired on the object-in-place task. Finally, the effect of MTT lesions on spatial working memory in the radial-arm maze was examined, to compare with a task that is known to be sensitive to MTT lesions (e.g., Vann, 2013; Vann & Aggleton, 2003) and requires animals to actively navigate within their environment. The radial-arm maze experiment included a manipulation, maze rotation, which selectively taxes the use of allocentric spatial information.[Fig-anchor fig1]

## Materials and Method

### Subjects and Surgery

Subjects were 23 male pigmented rats (Dark Agouti strain; Harlan, Bicester, United Kingdom) weighing between 226 g and 252 g at the time of surgery. Animals were housed in pairs under diurnal light conditions (14 hr light/10 hr dark) and testing was carried out during the light phase. Animals were given free access to water and a large cardboard tube and wooden chew-stick were available in the home-cage throughout. For all behavioral experiments, the animals were placed on a food restricted diet where they were still able to gain weight; their weights did not fall below 85% of their equivalent free feeding weight. All experiments were carried out in accordance with United Kingdom Animals (Scientific Procedures) Act, 1986 and associated guidelines.

Prior to surgery, all animals were deeply anesthetized by intraperitoneal injection of sodium pentobarbital (60 mg/kg pentobarbital sodium salt; Sigma-Aldrich, United Kingdom). The animals were then placed in a stereotaxic headholder (David Kopf Instruments, Tujunga, CA), with the nose-bar at +5.0, and a longitudinal incision was made in the scalp, which was retracted to expose the skull. The skull was drilled at the point of the lesion. The mammillothalamic tract lesions (MTTx; *n* = 13) were made by radiofrequency using a thermocouple radiofrequency electrode (0.7 mm active tip length, 0.25 mm diameter; Diros Technology Inc., Toronto, Canada). The electrode was lowered vertically and the tip temperature was raised to 70 °C for 22 s using an OWL Universal RF System URF-3AP lesion maker (Diros Technology Inc., Toronto, Canada). The stereotaxic coordinates for the lesions were: AP, −2.0; L, ±0.9 (both relative to bregma); and the depth, from top of cortex, was −6.2 mm. The surgical control rats (Sham; *n* = 10) underwent the same procedures except the probe was lowered to +1.0 mm above the lesion site; the temperature of the probe was not raised.

During surgery, rats were maintained on oxygen and given an analgesic (Meloxicam; Boehringer Ingelheim, Rhein, Germany). On completion of surgery, the skin was sutured and antibiotic powder (Clindamycin Hydrochloride, Pharmacia, Sandwich, United Kingdom) was applied topically to the wound site. Animals also received subcutaneous injections of 5 ml glucose saline. All animals recovered well following surgery.

Behavioral testing began 4 weeks following the completion of surgery. Each rat performed all behavioral tasks in the same sequential order. To avoid possible positive and negative transfer effects, control tasks (Experiment 1: nonspatial discrimination; Experiment 2: standard object recognition) were conducted prior to tasks that contained the critical spatial element.

## Experiment 1a: Nonspatial Discrimination

### Pretraining

During pretraining, two identical round digging cups (6.5 cm tall and 7 cm in diameter) made of black plastic were used to train rats to dig for food rewards. Each cup was filled with shredded paper, and a false bottom made of metal grille was inserted into the base to allow Cheerios (Nestle, United Kingdom) to be hidden beneath where they could be smelled by the rats but not accessed. The cups could be fixed with Velcro to the floor of the pretraining arena, a rectangular white plastic box measuring 25 cm × 42 cm with walls 12 cm high. The cups were placed 2 cm away from the short end wall of the arena. Habituation took place in a room measuring 300 cm × 285 cm with walls 255 cm high.

The MTTx and Sham rats were habituated to the digging cups and arena over 4 days, receiving 10 min in the apparatus each day. It has been shown previously that this is sufficient time for animals to learn to dig for rewards (Hindley, Nelson, Aggleton, & Vann, 2014). The cups were filled with sawdust mixed with crumbled Cheerios to mask the smell of a hidden Cheerio reward. One cup was placed at either end of the habituation arena. On the first day of habituation half a Cheerio was placed on top of the paper in either cup. To encourage exploration of both cups, each cup was only rebaited after the other Cheerio had been eaten. White noise, at approximately 75db, was present throughout habituation. The source of the white noise was directly underneath the digging arena. After the 4 days of pretraining, all rats had successfully learnt to dig to retrieve the reward.

### Test Procedure

#### Apparatus

Both digging cups were made of a square cup made of white plastic and measured 8 cm × 8 cm × 6 cm. To differentiate the two cups, one cup also had a band of black tape around it. A metal grille was inserted into the base of both cups to allow Cheerios to be hidden inside without being accessible to the rats. One cup was filled with small plastic beads (Hama Beads, Malta Haaning Plastic A/S, Denmark) and the round cup with shredded paper. The cup was fixed with Velcro onto the floor of the test arena, a rectangular white plastic box measuring 25 cm × 42 cm with walls 12 cm high. The cup was placed 2 cm away from one end of the arena, and was changed each time the digging medium changed. The arena was placed on a table measuring 110 cm × 55 cm × 76 cm, and against one wall of a room measuring 300 cm × 285 cm × 240 cm. White noise (75db) was played throughout training.

#### Procedure

Rats were presented with a digging cup filled with either shredded paper or Hama beads, and were rewarded for digging in one medium but not the other. The rewarded medium was counterbalanced across groups. Rats were placed in the arena at the opposite end to the digging cup, and the time taken to start digging measured. During a rewarded (“go”) trial, rats that did not dig were shown the Cheerio. Rats were removed from the arena 5 s after finding the reward. During unrewarded no-go trials, the rat was removed from the arena after 20 s if it had not dug or was removed after it had finished digging and found no reward. Digging was defined as breaking the surface of the digging medium with the nose or paws. The latency to dig was recorded for each trial and a difference score calculated, that is, dig latency on rewarded (go) trials minus dig latency on nonrewarded (no-go) trials. A positive score was, therefore, evidence of discrimination between the rewarded and nonrewarded pots and a score of zero would indicate no discrimination. Rats were given 16 spaced trials per day in groups of four animals (eight rewarded and eight nonrewarded trials, pseudorandom trial order with a maximum of two consecutive trials of the same type), giving an intertrial interval of approximately 90 s. The rats were trained for 2 days on the nonspatial discrimination.

## Experiment 1b: Location Discrimination

### Apparatus

Testing took place in a novel room which measured 340 cm × 338 cm × 240 cm. One of the round black-plastic cups (from pretraining) was used for the location discrimination. The test arena consisted of an opaque plastic box measuring 36 cm × 25 cm with walls 10.5 cm high. There were salient visual cues on the walls, such as posters and shapes made from colored paper, to differentiate the different areas of the room. Two identical tables, 55 cm × 47 cm × 77 cm high, were placed in diagonally opposite corners of the room. The digging arena was placed on one of these tables for each trial. The cup was filled with sawdust. The light level in the center of the arena was 400 lux.

### Procedure

**Figure 1 fig1:**
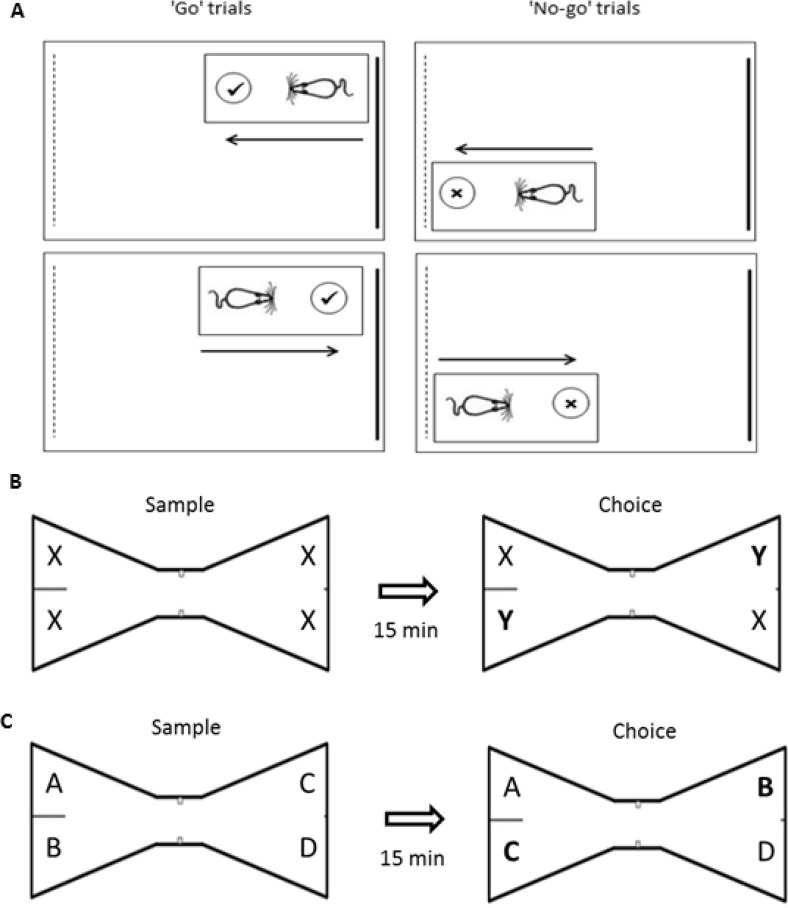
**A**: Location discrimination. Rats were required to discriminate one corner of a room from another. Rats were rewarded for digging in the correct (“go” trials), but not incorrect (“no-go” trials) corner. Direction of travel was irrelevant to solving the task. The schematic is not to scale. **B**: Standard object recognition. During the sample phase, rats explored four identical objects (X). In the choice phase, two of the familiar objects were replaced with a pair of novel objects (Y). Successful object recognition is demonstrated by preferential exploration of the novel (Y) over the familiar (X) objects. **C**: Object in place. During the sample phase, rats explored four different objects, each in a unique spatial location. In the choice phase, two of the objects (B/C) had swapped spatial location and the remaining two objects (A/D) had not been displaced. Successful object in place recognition memory is demonstrated by preferential exploration of the displaced (B/C) over the nondisplaced objects (A/D).

This experiment required rats to discriminate between two distinct locations within a room. As this experiment directly followed Experiment 1a, no habituation took place. There was only one test arena, which was moved between the two identical tables located in diagonally opposite corners of the room. Using the same arena throughout reduced the likelihood of the animals’ using any local odor cues to discriminate location. To reduce odor cues further, the arena was cleaned with alcohol wipes after each session (the completion of a day’s testing for four rats whose trials are interleaved with each other). Rats were rewarded for digging when in one arena location (go trials) but were not rewarded for digging in the other location (no-go trials), with the correct location counterbalanced across groups. Rats were not rewarded for making a correct response on “no-go” trials, that is, they were not rewarded for inhibiting the dig response when in the incorrect corner. Rats were placed in the arena so that they had to approach the cup from one of two directions in each location and could not use direction per se as a discriminative cue (see Figure 1A). Background white noise was played throughout training. Rats completed 16 trials a day (eight rewarded and eight nonrewarded), each trial lasting a maximum 20 s. Trial order (rewarded vs. nonrewarded) was pseudorandom with the only stipulation that there were no more than two consecutive trials of the same type. The rats were trained for 16 days. As in Experiment 1a, a difference score was calculated by subtracting the latency to dig on “go” trials from the latency to dig on “no-go” trials.

## Experiment 2: Standard Object Recognition and Object-in-Place

### Apparatus

Rats were tested in a “bowtie” maze, which had a wooden floor and steel walls, 50 cm high (Figures 1B/C; Albasser et al., 2010). The arena was placed on a table at a height of 76 cm, and against one wall of a room measuring 195 cm × 330 cm. The maze was 120 cm long, with two triangular ends 50 cm wide at their widest point. A corridor, 12 cm wide, joined the apices of these two triangles. The wide end of each triangle had two food wells recessed into the floor, 3.5 cm in diameter and 2 cm deep, which were covered by the objects being explored. Each of the objects used in this task was heavy enough that they could not be displaced by the rat, and were tall enough that the animals could not easily jump on top of them. The experiment used multiple duplicate sets of junk objects that were made of glass, metal, or plastic. Examples of objects used were glass bottles, plastic containers, and metal cans. On one side of the maze, the food wells were separated from each other by a steel dividing wall 48 cm high, which extended 15 cm from the middle of the back wall of the maze. To differentiate the two ends of the maze, the dividing wall was not present and a sheet of white bench-guard paper was attached to the maze wall at one end. A camera fixed to the ceiling above the maze was used to record onto DVD the animals’ object exploration for subsequent analysis.

### Procedure

Rats were brought to the testing room in individual metal carrying boxes, with a lid to prevent the animal from seeing outside the box. Prior to the test day, all animals were given two habituation sessions, during which the rat was placed in the arena for 10 min. No objects were present in the maze during habituation.

Thereafter, the rats underwent two tests of standard object recognition followed by two tests of object-in-place. A minimum of 48 hr separated each test session and different sets of objects were used in each test session.

### Standard Object Recognition

During the sample phase, one of four identical copies of the same object was placed on top of each of the wells in the “bowtie” maze. Rats were placed in the corridor of the maze and allowed to explore the objects for 5 min. Exploration was defined as time spent with the nose pointing toward the object at a distance of less than 1 cm. At the end of the sample phase the rat was removed from the maze and returned to the carrying box. During the 15-min intertrial interval, the arena was wiped down with a 20% ethanol solution to reduce odor cues and all objects from the sample phase were removed from the arena. Two objects (one object per end of the arena and diagonally opposite each other) were replaced with identical copies of the same object (familiar objects) and a new set of two identical objects (novel objects) was placed in the arena (one novel object per end of the arena and diagonally opposite each other) so that at each end of the arena there was one familiar and one novel object (see Figure 1B). The location of novel and familiar objects was counterbalanced across animals and sessions. Following the intertrial interval, the rat was returned to the arena for a 3 min test phase, during which the time spent exploring the familiar and novel objects was recorded. In order to assess any differential exploration of the novel and familiar objects as well as take account of each rats’ individual level of exploration, a discrimination ratio (D2) was calculated by dividing the difference in time spent exploring the novel and familiar objects by the total exploration of both sets of objects.

### Object-in-Place

During the sample phase, four different objects were placed in the maze, one on top of each of the wells in the “bowtie” maze. Rats were placed in the center of the maze to start the sample phase, and allowed to explore the objects for 5 min. At the end of the sample phase the rat was removed from the maze and returned to the carrying box. During the 15 min intertrial interval the maze was wiped down with 20% ethanol to reduce odor cues as far as possible, and the objects were removed and replaced with replicas of the original objects. Two of the objects, diagonally opposite each other, were interchanged so that they were now in different positions in the arena, while the other two objects remained in the same position as they had occupied during the sample phase. Following the intertrial interval, the rat was returned to the maze for a 3 min test phase, during which the time spent exploring the displaced and nondisplaced objects was recorded (see Figure 1C). The positions of the objects that were moved for the test phase were counterbalanced across sessions. A D2 discrimination ratio was calculated by subtracting the time spent exploring the displaced objects from the time spent exploring the nondisplaced object and dividing that difference by the total exploration time.

## Experiment 3: Radial-Arm Maze (Acquisition and Rotation)

### Apparatus

Testing was carried out in an eight-arm radial maze. The maze consisted of an octagonal central platform (34 cm diameter) and eight equally spaced radial arms (87 cm long, 10 cm wide). The base of the central platform and the arms were made of wood and panels of clear Perspex (24 cm high) formed the walls of the arms. At the start of each arm was a clear Perspex guillotine door (12 cm high) attached to a pulley. The maze was positioned in a room (255 × 330 × 260 cm) that contained salient visual cues such as geometric shapes and high contrast stimuli on the walls.

### Procedure

Pretraining involved two habituation sessions where the animals were allowed to explore the maze freely for 5 min with the guillotine doors raised and food pellets (45 mg; Noyes Purified Rodent Diet, U.K.) scattered down the arms. The animals were then trained on the standard radial-arm maze task (see below). A time limit of 10 min was placed on each session. Animals were tested until they had completed 18 sessions in Stage I and six sessions in Stage II.

Stage I (Sessions 1–18): At the start of a trial all eight arms were baited with two food pellets. The animal was allowed to make an arm choice and then return to the central platform. All the doors were closed for then about 10s before they were reopened, permitting the animal to make another choice. This procedure continued until all eight arms had been visited or 10 min had elapsed. Only trials where animals made a minimum of eight arm choices were included in the analyses.

Stage II (Sessions 19–24) tested for the possible use of intramaze cues when performing the task. The start of the session was as before but after the animal had made four different arm choices it was contained in the center of the maze while the maze was rotated by 45^o^ (clockwise/anticlockwise on alternate days). The remaining food pellets were moved so that they were still in the same allocentric room locations but the actual arms had changed; the experimenter pretended to bait each arm while moving the pellets so not to inadvertently cue the animal. The session then continued until all reward pellets had been retrieved.

### Histological Procedures

At the end of the behavioral experiments, the rats were deeply anesthetized with sodium pentobarbital (60 mg/kg, Euthatal, Rhone Merieux, United Kingdom) and transcardially perfused with 0.1-M phosphate buffer saline (PBS) followed by 4% paraformaldehyde in 0.1 M PBS (PFA). The brains were removed and postfixed in PFA for 4 hr and then transferred to 25% sucrose overnight at room temperature with rotation. Sections were cut at 40 μm on a freezing microtome in the coronal plane.

One series (one-in-four) of sections was mounted onto gelatin-coated slides and stained with cresyl violet, a Nissl stain, for histological assessment.

## Results

### Histological Analysis

**Figure 2 fig2:**
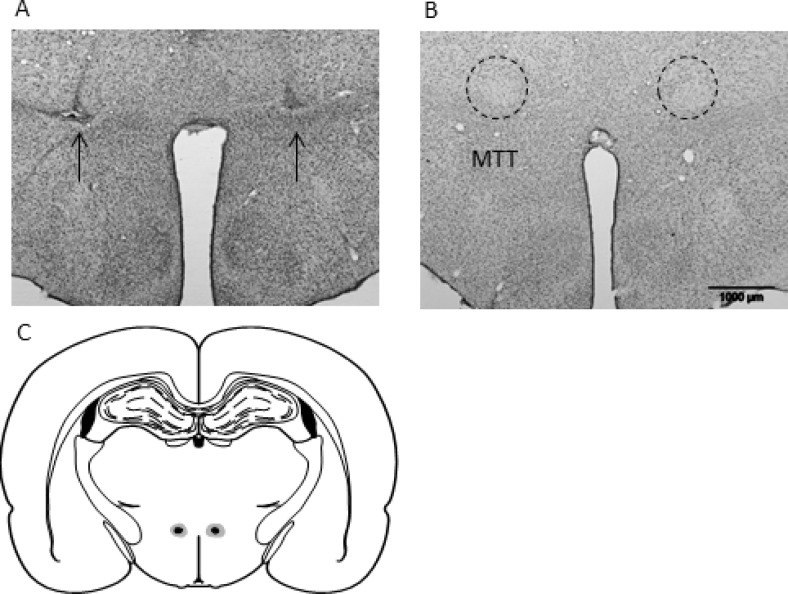
Photomicrographs depicting typical lesions. Nissl-stained section showing a mammillothalamic tract lesion (A) and surgical Sham (B), the arrows indicate the lesion. (C) Schematic reconstruction of a coronal section (Paxinos & Watson, 2007) showing the largest (light gray) and the smallest (black) mammillothalamic tract lesions (approximately −2.0 mm behind bregma).

Ten of the 13 MTTx rats had complete, bilateral lesions of the MTT. The remaining three cases had sparing in one or both hemispheres and were consequently removed from all subsequent analyses. All remaining cases involved discrete lesions of the MTT that were sufficiently anterior so the there was no direct damage to the supramammillary nuclei, the mammillary bodies, or the mammillotegmental tract. Similarly, the lesions did not encroach on the postcommissural portion of the fornix (see Figure 2). Final numbers: MTTx = 10; Sham = 10.[Fig-anchor fig2]

### Behavioral Results

#### Experiment 1a: Nonspatial discrimination

**Figure 3 fig3:**
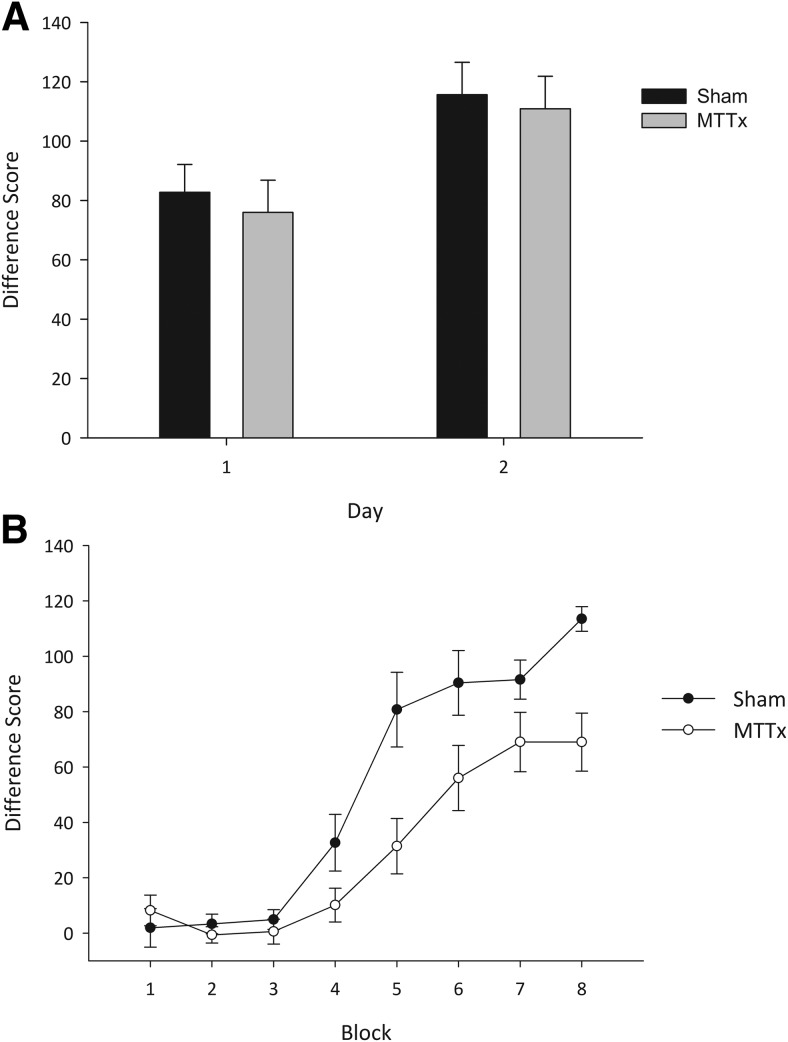
**A:** Nonspatial discrimination. The mean difference scores (±SEM) for the two groups across the two days of training are shown. The difference score represents the mean latency to dig on “go” trials subtracted from the mean latency to dig on “no-go” trials. Both groups readily discriminated the rewarded from the nonrewarded digging medium. **B**: Location discrimination. The mean difference scores (±SEM) for the two groups across the eight blocks of training on the location discrimination are shown. The difference score represents the mean latency to dig on “go” trials subtracted from the mean latency to do on “no-go” trials. Although both groups performed significantly above chance level by the end of training, the MTTx group acquired the location discrimination more slowly and less accurately than the Sham animals.

Both MTTx and Sham animals rapidly discriminated the digging media and were able to inhibit responding during nonrewarded trials as evidenced by differences scores that were above chance levels on both days (MTTx min *t*(9) = 8.1, *p* < .001; Sham min *t*(9) = 6.9, *p* < .001). As is clear from Figure 3A, there was no difference between the Sham and the MTTx animals. ANOVA yielded only an effect of day, as performance improved across the two days of training, *F*(1, 18) = 8.4, *p* < .01, but this trend was unaffected by lesion group as there no interaction with lesion or any lesion effect (*F*s < 1).[Fig-anchor fig3]

#### Experiment 1b: Location discrimination

Preliminary analysis of the mean latency to dig on “go” and “no-go” trials on Day 1 revealed no differences between the two groups (all *F*s < 1). As such, baseline differences in activity are unlikely to account for any group differences observed during training. For ease of presentation, the difference score is used in all subsequent analyses.

In stark contrast to Experiment 1a, the MTTx group was markedly impaired on the location discrimination when the animals were required to discriminate one corner of a room from another. As is clear from Figure 3B, as well as taking longer to acquire the location discrimination, the MTTx group performed the task overall less accurately than the Shams. ANOVA confirmed this description of the data as there was an effect of block, *F*(7,126) = 74.4, *p* < .001; a block by lesion interaction, *F*(7,126) = 5.3, *p* < .001; and an effect of group, *F*(1, 18) = 6.6, *p* < .05. One-sample *t* tests confirmed that Sham performance was consistently above chance levels from Block 4 (min *t*(9) = 3.2, *p* < .05), and the MTTx group did not perform above chance until Block 5 (min *t*(9) = 3.2, *p* < .05).

#### Experiment 2a: Object recognition

Exploration times during the sample phase did not differ by lesion group (F<1) [Mean s ± S.E.M: Shams = 54.9 ± 3.1; MTTx = 56.4 ± 4.8].

**Figure 4 fig4:**
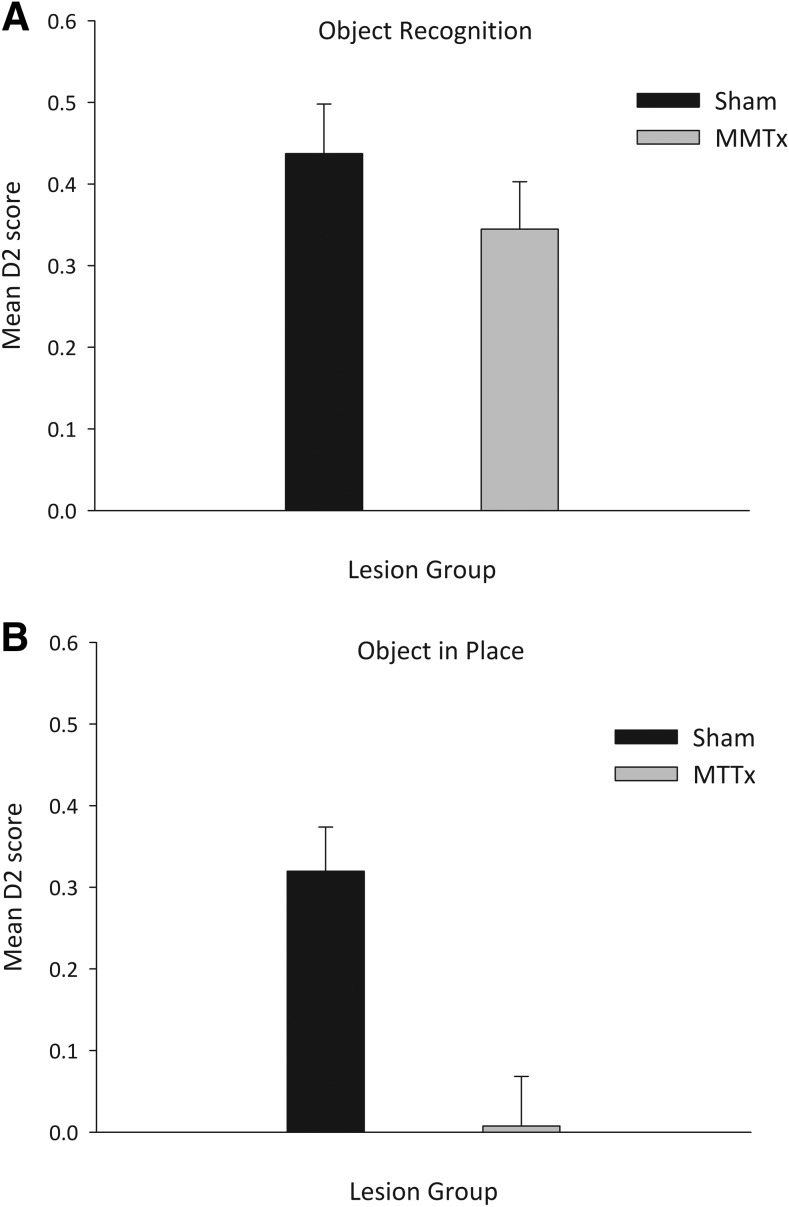
Object recognition memory and object-in-place memory. Performance of the Sham and MTTx groups on tests of object recognition memory (A) and object-in-place memory (B) expressed as mean D2 scores (±SEM). A score above 0 represents preferential exploration of the novel (A) or displaced (B) objects. MTT lesions selectively disrupted object-in-place memory which taxes animals’ ability to recognize a change in the spatial array of familiar objects.

Initial analysis revealed no difference in performance across the two test sessions (*F* < 1) so the data were collapsed across sessions. There was no difference between the two groups in overall exploration time at test (*F* < 1; Mean s ± S.E.M Shams = 42.4 ± 4.2; MTTx = 43.7 ± 3.4). Moreover, both Shams and MTTx showed intact object recognition memory as, at test, they preferentially explored the novel objects (Figure 4A). Both groups performed above chance levels (Shams *t*(9) = 7.2, *p* < .001; MTTx *t*(9) = 5.9, *p* < .001) and there was no difference between the two groups, *F*(1, 18) = 1.2, *p* = .29.[Fig-anchor fig4]

#### Experiment 2b: Object-in-place

Again, there were no differences in exploration between the two groups during the sample phases (*F* < 1; Mean s ± S.E.M: Shams = 59.9 ± 3.5; MTTx = 61.2 ± 2.9).

Test performance did not differ by session, *F*(1, 18) = 1.46, *p* = .24, so the data were collapsed across sessions. There was no difference between the groups in terms of the total exploration time during the test, *F*(1, 18) = 1.45, *p* = .24 (Mean ± S.E.M Shams = 42.4 s ± 3.0; MTTx = 36.9 s ± 3.4). However as is clear from Figure 4B, Shams spent more time exploring the objects in novel positions (*t*(9) = 5.9, *p* < .001) whereas the MTTx group did not respond preferentially to the displaced objects (*t* < 1). ANOVA confirmed an effect of group, *F*(1, 19) 14.8, *p* < .001, as the MTTx group had lower D2 scores than the Shams.

#### Experiment 3: Radial-arm maze (acquisition and rotation)

**Figure 5 fig5:**
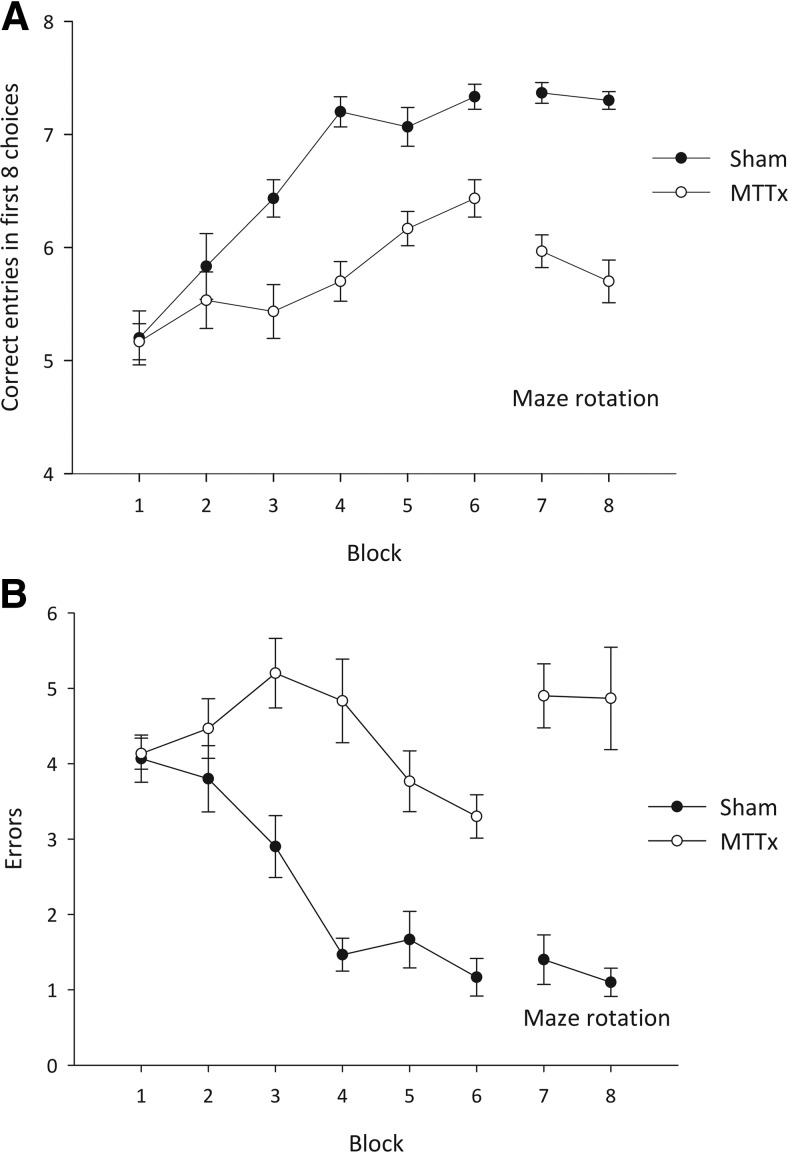
Radial-arm maze task. A. Mean number of correct entries in the first eight arm choices (±SEM). The first six blocks represent acquisition of the task and the final two blocks include maze rotation; B. Mean number of errors (±SEM) during acquisition (first six blocks) and rotation (final two blocks). Using both measures, the MTTx group was impaired relative to the Sham group during both acquisition and rotation.

Both total correct entries and number of errors during the acquisition (Stage I) and rotation (Stage II) phases were analyzed. As is clear from Figure 5A/B, during acquisition (Stage I), the MTTx made fewer correct entries, *F*(1, 18) = 27.4, *p* < .001, and made more errors than the Shams, *F*(1, 18) = 37.0, *p* < .001. Both performance measures improved over training, as there was an effect of block, min *F*(5, 90) = 11.6, *p* < .001, but the rate of improvement differed by group as there was a block by lesion interaction, min *F*(5, 90) = 4.8, *p* < .001.[Fig-anchor fig5]

In Stage II, to preclude the use of intramaze cues, the maze was rotated after the first four arm choices. The MTTx group performed significantly worse than the Shams, both in terms of the number of correct entries, *F*(1, 18) = 101.3, *p* < .001, and total number of errors, *F*(1, 18) = 51.0, *p* < .001, made. There was no effect of block nor block by lesion interaction using either measure, max *F*(1, 18) = 2.1, *p* = .17.

Subsequent analyses, comparing performance during the last two blocks of Stage I (standard condition) and the two blocks of rotation, revealed an interaction between test condition and lesion, *F*(1, 18) = 6.7, *p* < .05. Sham performance did not differ across the two conditions (*F* < 1) but the MTTx group made more errors when forced to rely on extramaze cues relative to the standard condition, *F*(1, 9) = 10.6, *p* < .01.

## Discussion

The aim of the current experiments was to explore further the nature of the spatial memory deficit associated with transection of the mammillothalamic tract (MTT). More specifically, to determine whether spatial deficits observed with MTT lesions were due to navigational components of the task or whether they could be found when navigational demands were limited. The results were clear cut: the MTT lesions consistently resulted in impairments on behavioral assays that taxed spatial memory, irrespective of the navigational aspect, while leaving performance intact on tasks that did not contain a spatial element.

In Experiment 1B, rats were required to discriminate two different locations in the same room and were rewarded for digging in the correct corner. Relative to Sham controls, the MTT lesion group acquired this go/no-go discrimination more slowly and less accurately, but nonetheless their performance was above chance levels by the fifth block of training. A control experiment showed that this impairment did not arise from nonspecific effects such as hyperactivity, general response perseveration, or deficits in attention and motivation that could potentially account for impaired go/no-go performance, as these animals were able to acquire a nonspatial go/no-go discrimination in which they were rewarded for digging in one media but not another (Experiment 1A). The location task contained no navigational component and there was no requirement for the animal to monitor where it had traveled within any given trial, as the task was explicitly designed so that direction of travel within the test room was irrelevant to solving the discrimination. Rather, animals had to rely on visual cues to discriminate the two corners of the room but the value of some of the cues will have been ambiguous, as both the rewarded and nonrewarded locations were associated with overlapping common cues. Thus, in order to solve the discrimination, rats had to ignore these common features and use proximity to multiple visual cues to dissociate the rewarded from the nonrewarded locations within the room. A previous set of experiments tested the effect of extensive mammillary body lesions on rats’ ability to link a spatial location with a specific visual cue. The animals were required to make an egocentric response (e.g., always turn left) in the presence of a single visual cue and make the opposite egocentric response (e.g., always turn right) in the presence of a different cue. Extensive lesions, which encompassed the medial and lateral mammillary bodies as well as the supramammillary nucleus, did not disrupt the acquisition of this task (Sziklas & Petrides, 2000; Sziklas, Petrides, & Leri, 1996). The apparent discrepancy between these previous findings and the ones reported here can, however, be reconciled. First, mammillary body lesions do not disrupt egocentric discriminations (e.g., Neave, Nagle, & Aggleton, 1997). Second, the demands of the current location discrimination were more complex, as rats could not use a single cue to discriminate between the two spatial locations but instead were required to learn the correct location with reference to an array of allocentric cues. The implication is that the discrete MTT lesions employed in the current study disrupted the flexible use of multiple spatial cues to determine the correct location within the testing environment. More broadly, the demonstration that MTT lesions retarded the acquisition of this location discrimination accords with the proposition that these lesions disrupt the effective use of allocentric spatial information and is consistent with the impairment in radial-arm maze performance seen in the current study (Experiment 3) as well as with the findings from previous experiments employing a variety of maze-based tasks (e.g., Field et al., 1978; Thomas & Gash, 1985; Vann, 2013; Vann & Aggleton, 2003). The current findings build on these previous reports in that they demonstrate that this deficit in the use of allocentric spatial information is present following MTT transection, even when the task is simplified by removing the navigational requirements as well as the need for the rat to monitor where it has traveled within any given trial. Indeed the results from the radial-arm maze task (Experiment 3) are consistent with the suggestion that MTT lesions lead to a spatial deficit that cannot be explained solely in terms of a navigational impairment, as maze rotation led to an increase in errors relative to performance under standard testing conditions (Figure 5B). Rotating the maze midway through the trials forces the animals to use extramaze cues to solve the task. If MTT lesions only resulted in a navigational impairment or a deficit in the monitoring of self-generated movement, it is not clear why maze rotation should produce any further detrimental effect on performance.

A notable feature of the results from Experiment 1 is that the MTT lesion-induced impairment was both relatively mild and transient, in that performance was consistently above chance from Day 5 and reached near-Sham levels by the end of training. This contrasts with the more profound and enduring deficit seen in the radial-maze task (Experiment 3) and elsewhere in other maze-based tasks (e.g., Vann, 2013; Vann & Aggleton, 2003). The relative mildness of the deficit observed on the location task in Experiment 1 may in part reflect the lack of a navigational component. However, it may also be due to the incremental nature of the task as, in contrast to tests of spatial working memory such as the radial-arm maze or matching-to-place in the water maze, effective performance did not depend on the rapid encoding of new spatial information on a trial by trial or session by session basis. Indeed, the current findings mirror those of a previous study which revealed a MTT lesion deficit on a spatial conditional discrimination that was most apparent during the initial stages of training (Vann, Honey, & Aggleton, 2003). In both these studies, the spatial information required to solve the discriminations remained constant across trials and sessions. The implication is that the MTT is necessary during the early stages of task acquisition, when novel spatial information is initially encoded, but, as training progresses and environmental cues remain unchanged, effective task performance can be supported by other neural systems. Similarly, the finding that MTT lesions did not preclude learning on this task presumably reflects the existence of multiple brain regions that can support spatial learning (Hindley et al., 2014).

In Experiment 2 we explicitly tested the proposition that behavioral tasks that require the rapid encoding of spatial information are particularly sensitive to the effects of MTT transection. Tests of object-in-place recognition memory assess reactivity to changes in the spatial array of objects (Dix & Aggleton, 1999). As such, this task taxes not only the ability to recognize a familiar item from a novel one (recognition memory), it requires the animal to encode the spatial location in which that object was first encountered. This information must be encoded rapidly and retained during the intertrial interval and then subsequently recalled at test. The MTT group was severely impaired on this task and did not perform above chance levels, as they were unable to discriminate the displaced from the nondisplaced objects. Significantly, standard object recognition memory was unaffected by MTT transection, indicating that the MTT group did not have a general impairment in recognition memory per se but rather was selectively sensitive to the spatial demands of the object-in-place task. Moreover, as the navigational demands of the two tests of recognition memory were matched, it is unlikely that the pattern of results observed on the object-in-place task can simply be attributed to impaired navigation. Rather, in light of the results from the location task in Experiment 1, the deficit may reflect an inability to discriminate the different locations within the maze.

The finding that MTT transection disrupts object-in-place recognition memory is perhaps not surprising given that lesions to the anterior thalamic nucleus in rats and monkeys (Parker & Gaffan, 1997a; Sziklas & Petrides, 1999; Wilton, Barid, Muir, Honey, & Aggleton, 2001) and the mammillary bodies in monkeys (Parker & Gaffan, 1997b) impair performance on a variety of tasks that require animals to link specific objects with specific spatial locations. Similarly, the demonstration here that MTT lesions had no discernible impact on the ability to recognize a familiar from a novel object is consistent with previous reports of spared object recognition memory following lesions to either the anterior thalamic nucleus (e.g., Moran & Dalrymple-Alford, 2003; Warburton & Aggleton, 1999; Wilton et al., 2001) or the mammillary bodies (e.g., Aggleton & Mishkin, 1985; Aggleton, Neave, Nagle, & Hunt, 1995). This apparent functional dissociation mirrors findings that patients with pathology to the mammillary bodies or MTT exhibit impaired recollection but spared familiarity on tests of recognition memory (e.g., Carlesimo et al., 2007; Tsivilis et al., 2008; Vann et al., 2009). Seen in a broader context, this evidence accords with the proposition that the mammillary body-anterior thalamic axis plays a preponderant role in recollective over familiarity-based recognition memory (e.g., Aggleton, Dumont, & Warburton, 2011).

In principle, the deficits observed here in tests of spatial memory could be ascribed to a loss of head-direction information. For example, salient visual cues are known to exert control over the preferred firing direction of head-direction cells (Goodridge & Taube, 1995). Moreover, the lateral mammillary bodies contain head-direction cells (Blair, Cho, & Sharp, 1998; Stackman & Taube, 1998) and the head-direction signal in the anterior thalamic nuclei is dependent on plasticity within this nucleus (Bassett, Tullman, & Taube, 2007; Blair et al., 1999). Although not directly assessed here, the lesions in the present study are most likely to have selectively disconnected the medial mammillary bodies from the anterior thalamic nucleus and spared many of the lateral mammillary body efferents. To index the extent of disconnection produced by MTT lesions, we have previously used anatomical retrograde tracing techniques and shown residual bilateral projections from the lateral mammillary bodies to the anterior thalamic after MTT lesions comparable with the ones reported here (see Vann & Albasser, 2009). Similarly, head-direction cells are found in several other brain regions including the postsubiculum, retrosplenial cortex, and enthorhinal cortex that would have been spared by the MTT lesions in the current study (Taube, 2007). Thus, it is unlikely that a loss of head-direction information can provide a complete account of the deficits seen in the current study. Support for this suggestion comes from recent findings showing that lesions comparable with the ones reported here do not impair the acquisition of a geometric task (Vann, 2013) that is known to be sensitive to the effects of lesions within the head-direction system (Aggleton, Poirer, Aggleton, Vann, & Pearce, 2009; Vann, 2011). Similarly, the spatial memory impairment associated with discrete lesions to the lateral mammillary bodies tends to be less pronounced and less enduring than the deficits induced by lesions to either the medial mammillary bodies or the MTT (Vann, 2005).

In summary, the current results showed that MTT lesions disrupt spatial performance even when there is little or no requirement for the animal to navigate through the environment or monitor self-movement cues. The present findings are also in accordance with previous data indicating that the MTT is particularly important for the rapid encoding of new spatial information and the effective use of allocentric spatial strategies, as deficits appear most pronounced during the early stages of task acquisition or when animals are forced to rely on allocentric information (e.g., Vann, 2013; Vann & Aggleton, 2003). These data add to an emerging appreciation of the importance of the mammillary bodies and their efferents via the MTT for mnemonic processes (e.g., Vann, 2010; Vann & Aggleton, 2004). Intriguingly, recent evidence has shown that this contribution to memory is largely independent of the hippocampal inputs to the mammillary bodies. Indeed, it appears that the mammillary bodies can still support spatial cognition in the absence of their subicular inputs, as lesions to the descending postcommissural fornix do not reproduce the effects of either MTT or mammillary body lesions on tests of spatial memory (Vann, 2013; Vann et al., 2011). Conversely lesions of the ventral tegmental nuclei of Gudden, the other principal input to the medial mammillary bodies, result in a remarkably similar profile of deficits on tests of spatial memory as is found after MTT lesions (Vann 2009, 2010, 2013). Similarly lesions to the dorsal tegmental nucleus of Gudden, which has reciprocal connections with the lateral mammillary bodies, have also been shown to impair tests of spatial memory (Clark et al., 2013; Dwyer et al., 2013). Thus, the functional significance of these parallel afferents from the limbic mesencephalon may prove critical to understanding the mammillary bodies’ role in memory (Vann, 2010; Vann, 2013; Vann & Aggleton, 2004).
